# Supplementary material for: A set of multi-entry identification keys to African frugivorous flies (Diptera, Tephritidae)
Source: Zookeys. 2014 Jul 24;(428):97–108. doi: 10.3897/zookeys.428.7366 (PMC4143993; doi:10.3897/zookeys.428.7366)
Supplement: Supplementary material 6 — Key to Ceratitis [file zookeys-428-097-s006.zip › SF6_ZooKeys_key to Ceratitis/key/SF6_ZooKeys_key to Ceratitis/Media/Html/Ceratitis nigricornis.htm]

Ceratitis (Pterandrus) nigricornis, n


***Ceratitis*** **(*Pterandrus*) *nigricornis* De Meyer & Freidberg**

Body
length: 4.30 mm; wing length: 4.20 mm.

Male

Head: Antenna black. First flagellomere 2.5 times as long as
pedicel. Arista with short rays; ventral rays shorter and sparser than dorsal
rays. Frons yellow, with brown irregular band widened in median part; with
short scattered setulae darker than frons. Face yellowish white, dorsal half
brownish black; gena with brown markings. Genal seta and setulae dark, well
developed.

Thorax: Postpronotal
lobe yellowish white, with black spot broadly connected to scutal pattern, or
almost so. Scutal pattern: ground color brownish black, microtrichose areas
silvery, prescutellar white markings separate but with paler brown area in
between. Scapular setae dark. Scutellum yellowish white, basally with two dark
spots broadly joined forming a basal band, apically with three merged black
spots (paratype shows indistinct separation), extending to basal spots and
touching. Anepisternum on ventral half brownish black; setulae pale except on
ventral half where black setulae on posterior half.

Legs:
Blackish except where otherwise noted; setation typical for subgenus, dark and
pale. Coxae and trochanters predominantly yellow to brown; base of femora, base
and tip of tibiae narrowly yellow to brown. Foreleg: femur swollen, posteriorly
with dispersed bush of short dark setulae along entire length, posterodorsal
setulae longer; ventral setae dark; tibia broadened. Midleg: femur ventrally
with dark feathering along distal 0.4, remaining with pale and sparse setulae;
tibia not feathered. Hindleg: femur at apical 0.25 with longer setulae dorsally
and ventrally.

Wing:
bands brownish. Marginal band forming continuous band with anterior part of
discal band; cubital band free; medial band absent; crossvein R-M just beyond
middle of discal cell. Apex of vein R_1_ extending slightly beyond
crossvein R-M. Crossvein DM-Cu oblique anterobasally.

Abdomen:
Mostly brown. Tergite 1 yellow along anterior half, shiny brownish black
posteriorly. Tergites 2 and 4 wholly with silvery band. Tergite 5 yellow with
narrow brown anterior and posterior margin, anterior margin with gap medially.
Male epandrium in lateral view with lateral surstylus slender and elongate;
posterior lobe straight.

Female

Unknown.

(Description
after De Meyer & Freidberg, 2006)
